# Anion Recognition by Pyrylium Cations and Thio-, Seleno- and Telluro- Analogues: A Combined Theoretical and Cambridge Structural Database Study

**DOI:** 10.3390/molecules200711632

**Published:** 2015-06-24

**Authors:** David Quiñonero

**Affiliations:** Departament de Química, Universitat de les Illes Balears, Crta. de Valldemossa km 7.5, 07122 Palma de Mallorca, Spain; E-Mail: david.quinonero@uib.es; Tel.: +34-971-173-498 (ext. 3498); Fax: +34-971-173-426

**Keywords:** chalcogen bond, anion-π interaction, noncovalent interactions, pyrylium complexes, anion recognition, DFT

## Abstract

Pyrylium salts are a very important class of organic molecules containing a trivalent oxygen atom in a six-membered aromatic ring. In this manuscript, we report a theoretical study of pyrylium salts and their thio-, seleno- and telluro- analogues by means of DFT calculations. For this purpose, unsubstituted 2,4,6-trimethyl and 2,4,6-triphenyl cations and anions with different morphologies were chosen (Cl^–^, NO_3_^–^ and BF_4_^–^). The complexes were characterized by means of natural bond orbital and “atoms-in-molecules” theories, and the physical nature of the interactions has been analyzed by means of symmetry-adapted perturbation theory calculations. Our results indicate the presence of anion-π interactions and chalcogen bonds based on both σ- and π-hole interactions and the existence of very favorable σ-complexes, especially for unsubstituted cations. The electrostatic component is dominant in the interactions, although the induction contributions are important, particularly for chloride complexes. The geometrical features of the complexes have been compared with experimental data retrieved from the Cambridge Structural Database.

## 1. Introduction

Noncovalent interactions have a constitutive role in the science of intermolecular relationships. In particular, those involving aromatic rings play a vital role in chemistry and biology [1], which becomes prominent in drug-receptor interactions, crystal engineering and protein folding [2]. In recent years, the contact between an anion and the region above the plane of an electron-deficient aromatic ring has also been recognized as a noncovalent bonding interaction. The nature of this interaction, designated an “anion-π bond” [3], has been described by numerous theoretical studies, which demonstrate that it is energetically favorable [3,4,5,6,7,8], in addition to several experimental investigations [9,10,11,12].

While anion-π interactions continue to gain attention as their role in chemical processes is being increasingly recognized [13,14,15,16,17,18], studies of cationic oxygenated aromatic rings involved in anion-π bonding were absent in the literature until very recently [19], where the authors report an experimental and theoretical study for pyrylium-based tri-arylated series of compounds with a tetrafluoroborate anion. Pyrylium salts are a very important class of cationic organic molecules containing a trivalent oxygen atom in a six-membered aromatic ring [20,21]. They can act as intermediates for a variety of syntheses [22] and have been exploited to design sensors for anions [23], amines, amino acids and chameleon labels for quantifying proteins [24], as well as a kind of ionic liquid crystal material, which can be used in the display industry [25,26].

**Scheme 1:**
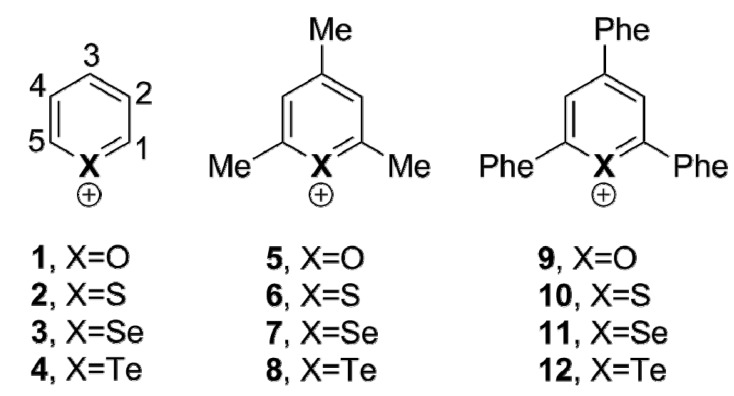
Molecules included in our study and the numbering of C atoms.

In this work, we present a systematic computational study on unsubstituted, trimethyl and triphenyl pyrylium cations (Scheme 1) interacting with Cl^–^, NO_3_^–^ and BF_4_^–^. We chose a monoatomic, a trigonal planar and a tetrahedral anion to account for different shapes and to analyze the effects that this would have in the formation of the complexes. Moreover, we also included in our study the thio-, seleno- and telluro- analogues of the pyrylium cations previously mentioned to analyze the effect of the chalcogen atom in such complexes (Scheme 1). The variation of the chalcogen atom comes also from the idea of studying the existence of chalcogen bonds. These noncovalent contacts belong to the σ-hole interaction family, as tetrel, pnicogen, halogen and aerogen bonds, which has attracted considerable attention in recent years, and they are recognized by the scientific community as powerful tools in supramolecular chemistry, crystal engineering and biochemistry [27,28,29,30,31,32]. We have also carried out natural bond orbital (NBO), atoms in molecules (AIM) and symmetry-adapted perturbation theory (SAPT) calculations to characterize the complexes and to analyze the physical nature of the interactions.

## 2. Results and Discussion

### 2.1. Preliminary Studies

**Figure 1:**
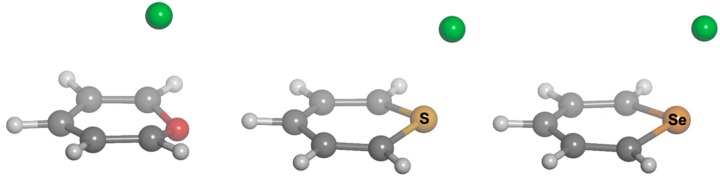
Optimized geometries of complexes of **1** (left), **2** (center) and **3** (right) with chloride.

We chose different aromatic systems to interact with chloride, namely pyrylium, thiopyrylium and selenopyrylium (**1**, **2** and **3**, respectively, in Scheme 1). We carried out Density Functional Theory (DFT) calculations at the BP86-D3/def2-TZVPD, B97-D3/def2-TZVPD levels and resolution of the identity (RI) second-order Møller Plesset (MP2) calculations at the RI-MP2/aug-cc-pVTZ (AVTZ) level. Geometry optimizations for all complexes. The geometries of the analyzed complexes are shown in Figure 1, and the geometric and energetic results of these calculations are gathered in Table 1. From the energetic point of view, regardless of the computational method used, all interaction energies are negative, indicating that the formation of the complexes between the aromatic systems and Cl^–^ is favorable, as expected from the interaction of two ions of opposite sign. The largest binding energy corresponds to the complex of **3**, which decreases for **2**·Cl^–^ and then again when the ring is **1**. Comparison of the DFT energy values yields larger binding energies for the BP86 than for the B97 results. The B97 interaction energies are in better agreement than the BP86 ones with the MP2 results, with energy differences as large as 2.7 kcal·mol^−1^. Furthermore, the agreement is even better when B97 and CCSD(T)/CBS (CCSD(T): coupled-cluster singles doubles and non-iterative triples correction; CBS: complete basis set) interaction energies are compared.

**Table 1:** Interaction energies (∆*E*, in kcal·mol^−1^) and equilibrium distances (R_e_, in Å) ^a^ of the complexes of Cl^–^ with **1**, **2** and **3** at the BP86-D3/TZVP, B97-D3/TZVP, MP2/AVTZ and CCSD(T)/CBS levels of theory.

Complexes	BP86-D3/TZVPD		B97-D3/TZVPD		MP2/AVTZ		CCSD(T)/CBS
	∆*E*	R_e_	∆*E*	R_e_	∆*E*	R_e_	∆*E*
**1**·Cl^–^	−103.2	2.90	−101.2	2.97	−98.9	2.86	−98.5
**2**·Cl^–^	−110.6	2.44	−106.4	2.50	−107.0	2.41	−104.6
**3**·Cl^–^	−112.9	2.52	−108.8	2.57	−111.5	2.49	−108.2

^a^ Distance between the Cl^–^ and X atom (see Scheme 1).

From the geometrical point of view, a comparison has also been made among the different levels of theory, with two points worth being mentioned. On the one hand, the equilibrium distances obtained at the B97 level are generally slightly longer (≥0.07 Å) than the ones obtained at the BP86 level (see Table 1). For instance, for the **1**· Cl^–^ complex, a 0.07 Å difference in length is observed. On the other hand, the BP86 equilibrium distances are in good agreement (better than the B97 ones) with the MP2 ones, where the former are slightly longer than the latter.

From the results gathered in Table 1, it can be deduced that despite the small differences in equilibrium distances, the B97 method nicely reproduces all of the interactions. Therefore, the comparison of B97 and CCSD(T) results validates the use of the RI-B97-D3/TZVP level of theory to study the interaction of our systems with Cl^–^. From now on, this is the computational methodology that will be used throughout the manuscript.

**Figure 2:**
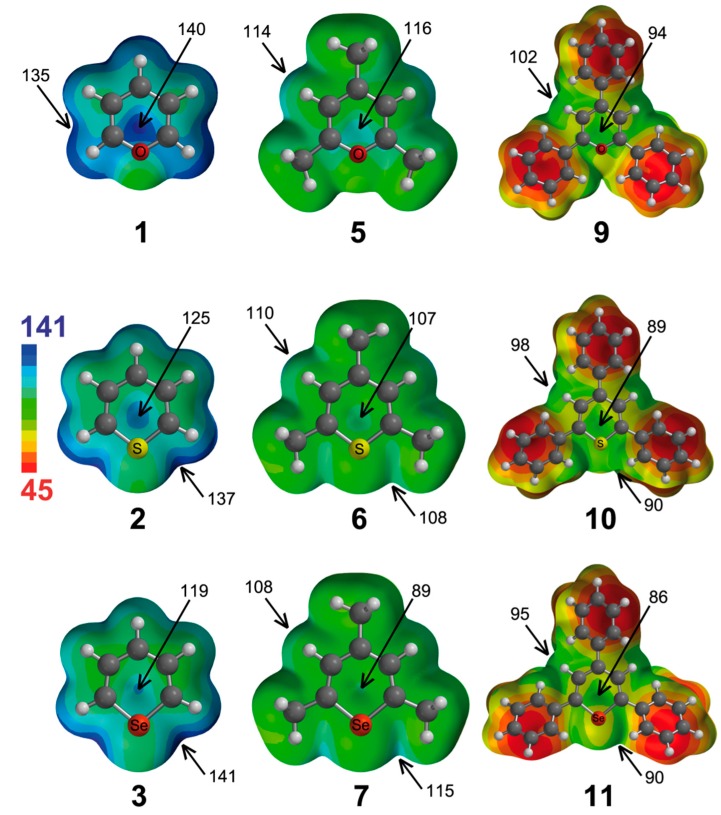
Molecular electrostatic potential surfaces of Molecules **1**–**3**, **5**–**7** and **9**–**11**. Some molecular electrostatic potential (MEP) energy values are indicated. Energies in kcal·mol^−1^.

We have also computed the molecular electrostatic potential (MEP) surfaces of the pyrylium cations and analogues (except from tellurium compounds), which are represented in Figure 2. From the analysis of the MEPs, several considerations can be made. The pyrylium Compound **1** has the highest electrostatic potential (ESP = 140 kcal·mol^−1^) located at the center of the molecule, although a little bit displaced towards the location of the oxygen atom. The ESPs associated with in-plane interactions are 5 kcal·mol^−1^ lower in energy. If we change the X atom, we observe how the ESP located above the molecular plane diminishes (to 125 and 119 kcal·mol^−1^ for **2** and **3**, respectively). Interestingly, the highest ESP for **2** and **3** is located at both sides of the X atom (137 and 141 kcal·mol^−1^, respectively). Therefore, from electrostatic considerations, the most favorable anion complex of **1** would be that where the anion is positioned above the ring, whereas the most favorable complexes of **2** and **3** would be those where the anion is located in the molecular plane and close to the X atom. However, for π interactions, polarization effects can be important, and the prediction based only on electrostatic effects may not be adequate. As inferred from Figure 2, the trimethyl Derivatives **5**–**7** have less positive values of ESP than their respective unsubstituted counterparts, and likewise, the triphenyl Analogues **9**–**11** have less positive ESP values than their respective trimethyl derivatives.

### 2.2. Complexes with Cl^–^

**Figure 3:**
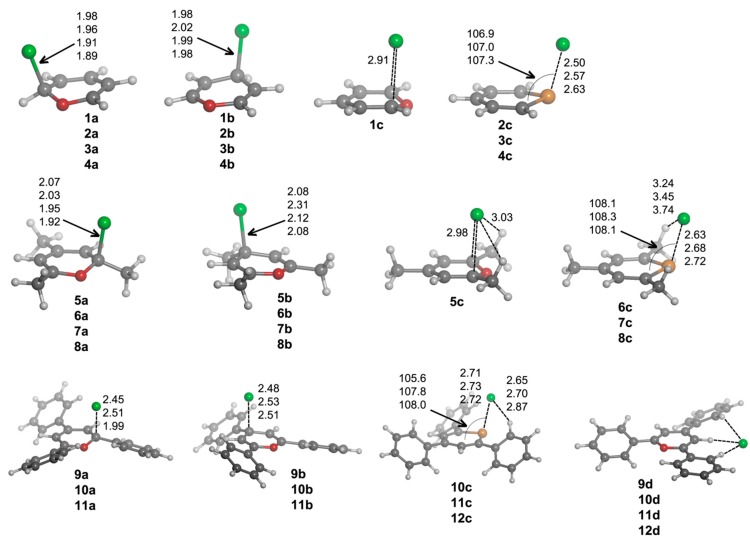
Optimized geometries of **a**·Cl^–^, **b**·Cl^–^, **c**·Cl^–^ and **d**·Cl^–^ complexes. Selected distances (in angstroms) and angles (in degrees) are shown.

First, we are going to analyze the complexes of Compounds **1**–**12** that are formed with chloride. After exploring the potential energy surface of the different complexes, four main structural configurations were found. Type **a** and **b** complexes are the result of a nucleophilic attack of the anion to C1 and C3 atoms, respectively, of the heteroaromatic ring, giving rise to σ-complexes. Thus, according to our nomenclature, **1a** will be the σ-complex of **1** with Cl^–^. In Type **c** complexes, the anion is located above the molecular plane, either interacting with C1 and C5 or the chalcogen atom. Lastly, Type **d** complexes are based on hydrogen bonding interactions. Several geometrical features of the complexes with chloride are shown in Figure 3, and their corresponding binding energies are gathered in Table 2.

From the inspection of the results, several points arise. For either **a** and **b** type complexes and within every family of compounds (unsubstituted, trimethyl and triphenyl substituted), the Cl–C distance is shortened when going down the chalcogen group. This result is consistent with the electrophilic character of the heteroaromatic ring, which is increased when going from O to Te atoms. Moreover, the Cl–C distance is increasingly lengthened when trimethyl and triphenyl substitution is considered, in agreement with the electrostatic results retrieved from MEP calculations (Figure 2). Second, **a** complexes have shorter Cl–C distances than **b** complex. Third, **12a**·Cl^–^ and **12b**·Cl^–^ adducts could not be located. All attempts to find such complexes led to **12c**·Cl^–^. For **c** complexes, we observe a slightly different placement of the anion if X = O (**1c**) or X = S, Se, Te (**2c**–**4c**) are considered. In Complex **1c**, the anion is located above the molecule and between C1 and C5, forcing the out-of-plane displacement of the oxygen atom opposite of Cl^–^. In fact, this complex is not a minimum on the potential energy surface, but a TS corresponding to the chloride transfer from the σ-complex on C1 (**1a**·Cl^–^) to its degenerate σ-complex on C5 [33]. However, for **2c**–**4c** complexes, Cl^–^ is parallel displaced towards the X atom, giving rise to a ClXC3 angle of *ca*. 107°. Moreover, for such complexes, the X atoms are pulled from the molecular plane towards the anion yielding Cl^–^···X distances (2.50, 2.57 and 2.63 Å for **2c**, **3c** and **4c**, respectively) that are remarkably much shorter than the sum of the van der Waals radii (3.91, 4.01 and 4.17 Å, respectively) [34,35]. In fact, all intermolecular contacts reported throughout the manuscript are substantially shorter than the sum of the corresponding van der Waals radii. Analogously to **a** and **b** complexes, the Cl^–^···X distance in **c** complexes is increasingly lengthened when trimethyl and triphenyl substitution is considered, which could be due, on the one hand, to the decrease in electrophilic character of the heteroaromatic ring for the trimethyl and triphenyl systems (Figure 2) and, on the other hand, to the presence of CH···Cl^–^ hydrogen bonds that slightly lower the net charge on the anion. These hydrogen bonds appear to be particularly important for the triphenyl derivatives, since their respective hydrogen bond distances are short (2.65–2.87 Å).

Every attempt to obtain exclusively hydrogen bond-based complexes was unsuccessful for **1**–**8** compounds, and the geometry optimizations always ended up yielding one of the geometries mentioned above. However, hydrogen bond complexes (Type **d**) were obtained for **9**–**12**, and their geometries are shown in Figure 3. All of these Type **d** complexes have the same recognition pattern, where the anion is interacting with two hydrogen atoms in ortho to two phenyl rings, with the hydrogen atom bonded to C2. The CH···Cl^–^ distance oscillates from 2.28–2.54 Å for X = O to 2.44–2.74 Å for X = Te.

**Table 2:** Interaction energies (**∆***E*, in kcal·mol^−1^), electron density (ρ_BCP_), its Laplacian (∇^2^ ρ_BCP_) and the total electron energy density (*H*_BCP_) for the bond critical points (BCPs) of Cl^–^···C or Cl^–^···X contacts of complexes of Compounds **1**–**12** with Cl^–^ and the number of imaginary frequencies (Nimag).

Complex	∆*E*	Nimag	ρ_BCP_ × 10^2^	∇^2^ ρ_BCP_ × 10^2^	*H*_BCP_
**1a**·Cl^–^	−116.0	0	12.43	−2.94	−5.55
**2a**·Cl^–^	−109.3	0	12.62	−3.11	−5.74
**3a**·Cl^–^	−110.9	0	13.98	−6.91	−7.10
**4a**·Cl^–^	−109.8	0	-	-	-
**1b**·Cl^–^	−115.2	0	11.94	−2.29	−5.26
**2b**·Cl^–^	−109.8	0	11.06	0.03	−4.48
**3b**·Cl^–^	−110.0	0	11.62	−1.35	−4.97
**4b**·Cl^–^	−107.9	0	-	-	-
**1c**·Cl^–^	−101.2	1	-	-	-
**2c**·Cl^–^	−106.4	0	6.10	8.47	−1.03
**3c**·Cl^–^	−108.8	0	5.76	7.97	−0.97
**4c**·Cl^–^	−114.0	0	-	-	-
**5a**·Cl^–^	−99.8	0	10.17	1.70	−3.65
**6a**·Cl^–^	−95.4	0	10.98	0.39	−4.30
**7a**·Cl^–^	−97.3	0	12.90	−4.30	−6.05
**8a**·Cl^–^	−97.6	0	-	-	-
**5b**·Cl^–^	−98.4	0	9.81	2.09	−3.49
**6b**·Cl^–^	−92.3	1	6.10	6.75	−1.10
**7b**·Cl^–^	−93.0	1	9.00	3.52	−2.87
**8b**·Cl^–^	−92.4	1	-	-	-
**5c**·Cl^–^	−93.6	1	-	-	-
**6c**·Cl^–^	−95.5	1	4.73	8.19	−0.51
**7c**·Cl^–^	−97.1	1	4.69	7.84	−0.52
**8c**·Cl^–^	−101.3	2	-	-	-
**9a**·Cl^–^	−84.9	2	-	-	-
**10a**·Cl^–^	−83.1	1	-	-	-
**11a**·Cl^–^	−84.2	1	-	-	-
**9b**·Cl^–^	−82.9	1	-	-	-
**10b**·Cl^–^	−83.7	0	-	-	-
**11b**·Cl^–^	−83.9	2	-	-	-
**10c**·Cl^–^	−88.7	0	4.12	7.46	−0.33
**11c**·Cl^–^	−91.0	0	4.24	7.32	−0.37
**12c**·Cl^–^	−96.3	0	-	-	-
**9d**·Cl^–^	−83.0	2	-	-	-
**10d**·Cl^–^	−77.5	0	-	-	-
**11d**·Cl^–^	−80.7	0	-	-	-
**12d**·Cl^–^	−79.2	0	-	-	-

The interaction energies of all complexes are large and negative, as shown in Table 2, ranging from −116.0 kcal·mol^–1^ for **1a**·Cl^–^ to −77.5 kcal·mol^–1^ for **10d**·Cl^–^, values than can be rationalized from the electrostatic point of view. The largest binding energies are generally found for **a** and **b** σ-complexes: For the unsubstituted compounds, we usually obtain the largest binding energies for **a** complexes. Analogously, the same trend is also observed for the trisubstituted derivatives, *i.e*., **a** complexes have larger ∆*E* values than **b** complexes, with **c** complexes having the smallest binding energies. However, we observe that the energy gap between **a** and **c** complexes gets smaller when going from **1** (∆∆*E* = 4.8 kcal·mol^−1^) to **3** (∆∆*E* = 0.9 kcal·mol^−1^). In fact, for Compound **4**, its **c** complex is more favorable than its **a** complex by 5.2 kcal·mol^−1^, leading to the conclusion that the more electrophilic the X atom, the more favorable the **c** complex. A similar behavior is observed for complexes of **5**–**8**, with ∆∆*E* values between **a** and **c** complexes of 6.2, 0.2 and −3.7 kcal·mol^−1^ for **1**, **3** and **4**, respectively. Strikingly, for Compounds **10**–**12**, their **c** complexes are the most favorable (neither **9c**·Cl^–^ nor **12a**·Cl^–^ could be located). For instance, the energy gap between **a** and **c** chloride complexes is −5.6 and −6.8 kcal·mol^−1^ for **10** and **11**, respectively.

**Figure 4:**
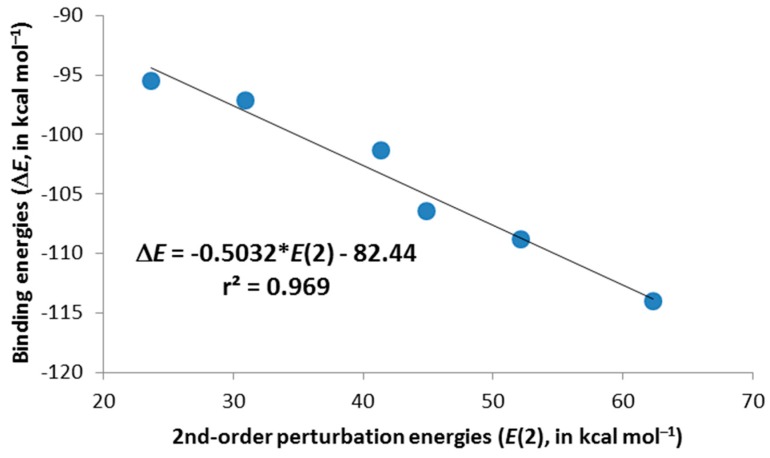
Selected second-order perturbation energies, *E*(2), plotted *vs.* the computed binding energies, ∆*E*, for **2c**–**4c**·Cl^–^ and **6c**–**8c**·Cl^–^ complexes.

To investigate the origin and nature of these interactions, AIM, NBO and SAPT studies have been carried out for selected complexes. We have examined all possible intermolecular interactions between occupied (donor) Lewis-type NBOs and vacant (acceptor) non-Lewis NBOs and estimated their energetic importance by second-order perturbation theory. According to the NBO analysis, the interaction in **c** complexes of X = S, Se and Te is primarily based on a charge donation from the lone pairs of Cl^–^ to the vacant π* orbital of the X–C1 or X–C5 bond, as derived from the calculated second-order orbital perturbation energies listed in Table 3. As a matter of fact, these charge-transfer contributions are very large, with increasing energies as we move from the lightest (44.9 kcal·mol^−1^ for **2c**·Cl^–^) to the heaviest (62.3 kcal·mol^−1^ for **4c**·Cl^–^) chalcogens for the unsubstituted complexes. The same trend is observed for the trimethyl derivatives, although with smaller energy contributions than those for their respective unsubstituted cases. Moreover, there is an excellent linear correlation (*r*^2^ = 0.969) between the binding energies (∆*E* in Table 2) and the second-order orbital perturbation energies (*E*(2) in Table 3) mentioned above for **2c**–**4c**·Cl^–^ and **6c**–**8c**·Cl^–^ complexes, indicating that these Lp(Cl^–^)→π*(X–C1/C5) donor-acceptor contributions are very relevant (Figure 4). It is worth mentioning the existence of other charge-transfer contributions of up to 3.7 kcal·mol^−1^ that arise from the charge donation from Cl^–^ lone pairs to empty d orbitals of S, Se and Te (Table 3). All of these results suggest the existence of a chalcogen bond through π-hole interactions. Differently from **2c**–**4c**·Cl^–^ and **6c**–**8c**·Cl^–^ complexes, there is no charge transfer from Cl^–^ to either O–C1 or O–C5 empty π* orbitals in **1c**·Cl^–^ and **5c**·Cl^–^. Instead, modest energetic contributions are obtained from Lp(Cl^–^)→π*(C1–C2) and Lp(Cl^–^)→π*(C4–C5) donor-acceptor interactions, leading to anion-π interactions. The absence of charge transfer processes in **1c**·Cl^–^ and **5c**·Cl^–^ indicates that induction forces are not important in their binding mechanism, which will be primarily based on electrostatics, as we will see later in the SAPT analysis. As already noted, the geometries of X = O complexes are different from the X = S, Se and Te ones (X is moving away with respect to the anion for the former and getting closer to the anion for the latter), giving rise to a different binding mechanism, where induction forces are supposed to play an important role in the latter compounds.

**Table 3:** Natural bond orbital (NBO) second-order orbital perturbation energies (*E*(2), in kcal·mol^−1^) derived from charge donation from an occupied (donor) to an empty (acceptor) orbital for Complexes **1c**–**8c**·Cl^–^.

Complex	Donor	Acceptor	*E*(2)
**1c**·Cl^–^	Lp(Cl^–^)	π*(C1–C2)	4.5
		π*(C4–C5)	4.5
**2c**·Cl^–^	Lp(Cl^–^)	π*(S–C1)	44.9
		Ry*(S)	3.7
**3c**·Cl^–^	Lp(Cl^–^)	π*(Se–C5)	52.1
		Ry*(Se)	2.3
**4c**·Cl^–^	Lp(Cl^–^)	π*(Te–C1)	62.3
			2.6
**5c**·Cl^–^	Lp(Cl^–^)	π*(C1–C2)	3.8
		π*(C4–C5)	3.8
**6c**·Cl^–^	Lp(Cl^–^)	π*(S–C5)	23.7
		Ry*(S)	2.7
**7c**·Cl^–^	Lp(Cl^–^)	π*(Se–C5)	30.9
		Ry*(Se)	1.6
**8c**·Cl^–^	Lp(Cl^–^)	π*(Te–C5)	41.4
		Ry*(Te)	2.3

**Figure 5:**
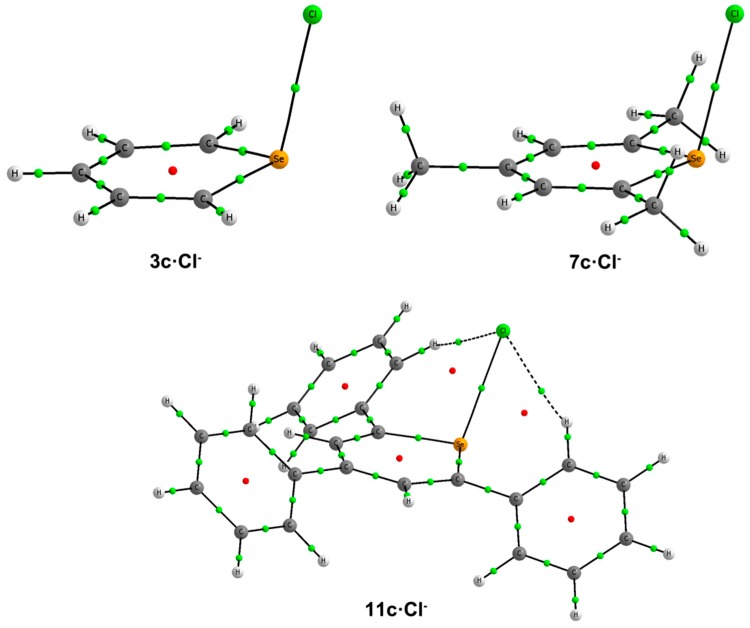
Molecular graphs of **2c**, **7c** and **11c**·Cl^–^. The BCPs and ring critical points are represented by green and red dots, respectively. Only bond paths are depicted.

In line with the NBO results, our AIM calculations for **2c**–**3c**·Cl^–^, **6c**–**7c**·Cl^–^ and **10c**–**11c**·Cl^–^ complexes show the existence of one BCP connecting the X atom with Cl^–^ (Figure 5). The values of the electron densities at these BCPs range between 0.061 au for **2c**·Cl^–^ and 0.041 au for **10c**·Cl^–^ (Table 2). At these BCPs, the values of the Laplacian are positive, and the total energy densities are negative (between −0.010 and −0.003 au), suggesting that these interactions have a certain covalent character [36].

In addition, the nature of the chemical bond in **1a**–**7a**·Cl^–^ and **1b**–**7b**·Cl^–^ σ-complexes has been analyzed by means of the AIM approach. From the inspection of the results (Table 2), the values of the electron density at the BCP originating from the Cl^–^ ···C1/C3 contact are large (between 0.14 and 0.06 au) with values of the Laplacian oscillating between negative (**1a**–**3a**, **7a**, **1b** and **3b**) and positive (**5a**–**6a**, **2b** and **5b**–**7b**). However, the total energy density for all cases is negative (between −0.071 and −0.011 au; Table 2), indicating that there is a significant sharing of electrons for this contact.

The physical nature of the interaction between the anion and Compounds **1**–**3**, **5**–**7** and **9**–**11** has been analyzed by means of SAPT calculations in **c** complexes. The energy contributions obtained from the SAPT partitioning scheme are listed in Table 4. As expected, the electrostatic component, *E*_el_, is the largest contributor to the interaction with values ranging from −103.8–−138.1 kcal·mol^−1^. The *E*_el_ values vary according to the electronegativity of the X atom, *i.e*., the smallest and largest contributions are found for X = O and X = Se, respectively. The positive exchange part is large and partly compensates for the electrostatic component, especially for X = S, Se complexes. The dispersion contribution is more or less constant for S and Se complexes and larger than that for O complexes. The induction-polarization contribution, *E*_ind_, however, seems to play an important role in the interaction, since large variations are detected, in line with the donor-acceptor NBO results. Thus, *E*_ind_ is small for the oxygen-based **1c** and **5c**·Cl^–^ complexes (*ca*. −12 kcal·mol^−1^), large for sulfur-based **2c**, **6c** and **10c**·Cl^–^ complexes (from −24 to −34 kcal·mol^−1^) and the largest for selenium-based **3c**, **7c**, and **11c**·Cl^–^ complexes (from −34–−48 kcal·mol^−1^). The consideration of higher-order contributions to the interaction energy, δHF (Table 4), is mandatory at least for X = S compounds to get a good agreement between SAPT interaction energies, *E*_SAPT_, and the binding energies.

**Table 4:** DF-DFT-symmetry-adapted perturbation theory (SAPT) electrostatic, exchange, induction, dispersion and Hartree-Fock higher-order energy contributions (*E*_el_, *E*_exch_, *E*_ind_, *E*_dis_ and δHF, respectively), SAPT interaction energy (*E*_SAPT_) and binding energies (∆*E*) of selected complexes. Energies in kcal·mol^−1^.

Complex	*E*_el_	*E*_exch_	*E*_ind_	*E*_disp_	δHF	*E*_SAPT_	∆*E*
**1c**·Cl^–^	−112.0	38.6	−12.2	−10.5	−4.6	−100.7	−101.2
**2c**·Cl^–^	−132.7	89.8	−34.5	−14.1	−10.0	−101.6	−106.4
**3c**·Cl^–^	−138.1	93.6	−48.0	−14.0	−2.1	−108.6	−108.8
**5c**·Cl^–^	−104.7	36.7	−12.1	−11.9	−2.2	−94.3	−93.6
**6c**·Cl^–^	−114.7	67.9	−24.4	−13.4	−6.0	−90.5	−95.5
**7c**·Cl^–^	−119.8	74.1	−34.4	−13.4	−1.3	−94.8	−97.1
**10c**·Cl^–^	−103.8	66.8	−25.6	−15.9	−6.0	−84.6	−88.7
**11c**·Cl^–^	−110.3	74.6	−34.9	−16.1	−2.3	−89.0	−91.0
**2c**·NO_3_^–^	−105.4	49.3	−14.0	−11.9	−7.3	−89.2	−96.4
**3c**·NO_3_^–^	−111.2	57.5	−19.3	−12.5	−8.0	−93.4	−97.3
**5e**·NO_3_^–^	−93.2	26.8	−7.1	−14.0	−2.8	−90.4	−90.9
**6e**·NO_3_^–^	−91.0	26.7	−7.6	−14.2	−2.8	−89.0	−89.0
**7e**·NO_3_^–^	−89.8	26.4	−7.8	−14.3	−2.8	−88.3	−88.7
**11c_1_**·NO_3_^–^	−80.0	32.1	−13.5	−13.9	−3.6	−78.9	−82.3
**12c_1_**·NO_3_^–^	−81.3	34.8	−14.6	−13.9	−3.9	−79.0	−83.8
**1e**·BF_4_^–^	−96.3	26.2	−6.5	−10.0	−2.8	−89.4	−89.3
**2e**·BF_4_^–^	−91.3	24.0	−6.5	−9.8	−2.5	−86.2	−85.8
**3e**·BF_4_^–^	−90.4	24.6	−6.8	−9.9	−2.7	−85.2	−84.9
**1a**·BF_4_^–^	−94.7	29.9	−7.8	−9.1	−4.8	−86.5	−87.2
**2c**·BF_4_^–^	−88.2	24.4	−8.8	−8.4	−3.6	−84.6	−84.7
**3c**·BF_4_^–^	−91.4	27.2	−10.0	−9.0	−4.0	−87.2	−86.8
**5e**·BF_4_^–^	−88.6	22.7	−7.2	−11.1	−1.9	−86.1	−85.6
**6e**·BF_4_^–^	−85.2	21.6	−7.3	−10.8	−1.7	−84.9	−82.9
**7e**·BF_4_^–^	−85.6	22.0	−7.6	−11.4	−1.8	−84.3	−82.3
**10c_2_**·BF_4_^–^	−73.9	23.0	−11.2	−12.6	−2.1	−76.8	−74.8
**11c_2_**·BF_4_^–^	−73.3	23.2	−11.3	−12.3	−2.3	−76.0	−74.1

### 2.3. Complexes with NO_3_^–^

**Figure 6:**
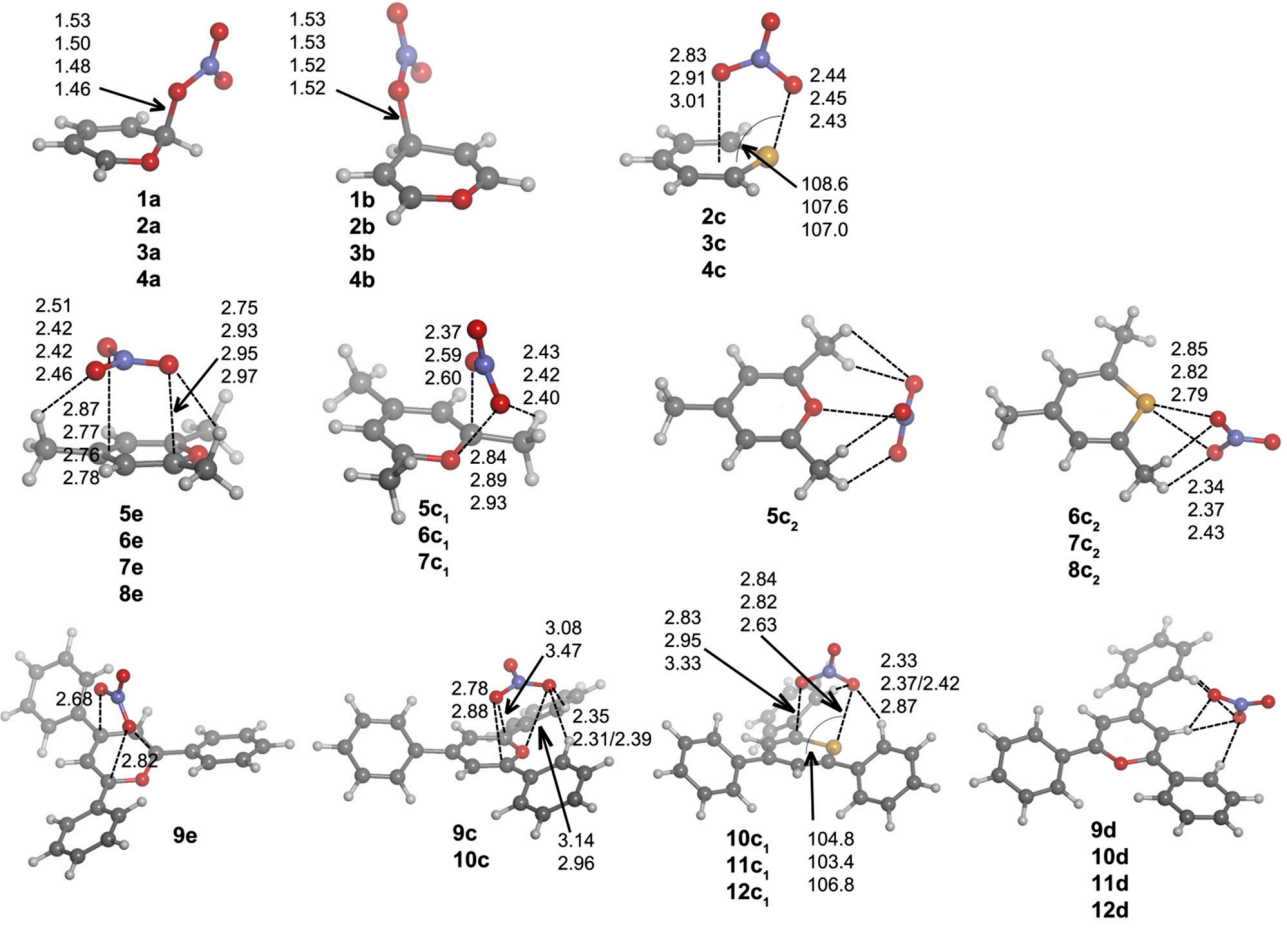
Optimized geometries of **a**·NO_3_^–^, **b**·NO_3_^–^, **c**·NO_3_^–^, **d**·NO_3_^–^ and **e**·NO_3_^–^ complexes. Selected distances (in angstroms) and angles (in degrees) are shown.

In this second section, we are going to analyze the complexes formed by Compounds **1**–**12** with nitrate as the anion. The exploration of the potential energy surface leads to the formation of five main structural configurations: Type **a**, **b** and **d** complexes, just as defined in the previous subsection, Type **c** complexes, which are very similar to the ones found for chloride, but now the anion is interacting either with the chalcogen atom or a C atom and the chalcogen atom, and the new Type **e** complexes, where the anion is solely interacting with C atoms above the molecular heteroaromatic ring. The geometries of the complexes can be found in Figure 6, and their respective binding energies are listed in Table 5.

Complexes **a** and **b** follow the same trend observed for chloride complexes, *i.e*., the O_2_NO–C distance shortens/lengthens with the size of the chalcogen atom/with trisubstitution, and **a** complexes have shorter O_2_NO–C distances than **b** complexes. However, **a** and **b** complexes are only obtained for the unsubstituted Compounds **1**–**4**. In **c**·NO_3_^–^ complexes, different orientations of nitrate are observed. For unsubstituted compounds, the nitrate is perpendicularly placed above the heteroaromatic ring, giving rise to short O_2_NO^–^···X contacts in **2c**–**4c**·NO_3_^–^ (Figure 6), similarly to what is observed for **2c**–**4c**·Cl^–^. Type **c** complexes for the trimethyl derivatives fall into two subcategories, depending on whether the anion is almost perpendicularly placed above, **c_1_**, or not, **c_2_**, the ring. In **5c_1_**–**7c_1_** complexes, the anion is slightly displaced towards the C1 half of the aromatic system, yielding three short contacts, as inferred from the distances between one O atom of nitrate with both an X and an H atom of the C1 methyl group and between another O atom of NO_3_^–^ with C1, as shown in Figure 6. The **c_1_** complex of **8** could not be found. In **5c_2_**–**8c_2_** complexes, the anion presents to different binding modes: all three O atoms of nitrate in **5c_2_** are interacting with two H atoms of C1 and C5 methyl groups, keeping the N atom sufficiently close to the O atom of the ring to expect a certain interaction. Conversely, in **6c_2_**–**8c_2_** complexes, two O atoms of the anion are equidistant to the X atom. In addition, these two O atoms are engaged in equidistant hydrogen-bonding interactions with each O atom with one H atom of the C5 methyl group. In triphenyl **c** complexes, two different NO_3_^–^ orientations are observed: those similar to the unsubstituted ones, **9c**–**10c**, and those similar to the trimethyl **c_1_** complexes, **10c_1_**–**12c_1_**. The **c** complexes of **11** and **12** and the **c_1_** complex of **9** could not be found. The main difference between **c** and **c1** complexes resides in the contacts between an O atom of nitrate and C1 and/or C5 atoms of the heteroaromatic system (Figure 6). Furthermore, the intermolecular O_2_NO^–^···X contacts are shorter for **c1** than for **c** complexes. All of these **c** and **c_1_** complexes present CH···ONO_2_^–^ hydrogen bonds with the H atoms in ortho to the C1 and C5 phenyl moieties.

**Table 5:** Interaction energies (∆*E*, in kcal·mol^−1^), electron density (ρ_BCP_), its Laplacian (∇^2^ ρ_BCP_) and the total electron energy density (*H*_BCP_) for the BCPs of O_2_NO^–^···C contacts of complexes of Compounds **1**–**12** with NO_3_^–^ and the number of imaginary frequencies (Nimag).

Complex	∆*E*	Nimag	ρ_BCP_ × 10^2^	∇^2^ ρ_BCP_ × 10^2^	*H*_BCP_ × 10^2^
**1a**·NO_3_^–^	−108.9	0	20.58	−28.06	−23.62
**2a**·NO_3_^–^	−103.0	0	21.79	−33.63	−21.21
**3a**·NO_3_^–^	−105.3	0	22.87	−39.79	−24.12
**4a**·NO_3_^–^	−104.8	0	-	-	-
**1b**·NO_3_^–^	−109.0	0	20.24	−26.26	−18.53
**2b**·NO_3_^–^	−102.8	0	19.97	−24.49	−17.91
**3b**·NO_3_^–^	−103.0	0	20.29	−26.19	−18.63
**4b**·NO_3_^–^	−100.9	0	-	-	-
**2c**·NO_3_^–^	−96.4	0	4.42	9.92	−0.31
**3c**·NO_3_^–^	−97.3	0	4.64	9.89	−0.39
**4c**·NO_3_^–^	−100.1	0	-	-	-
**5e**·NO_3_^– a^	−90.9	0	1.47	4.89	0.18
**6e**·NO_3_^– a^	−89.0	0	1.04	3.83	0.18
**7e**·NO_3_^– a^	−88.7	0	1.00	3.67	0.18
**8e**·NO_3_^–^	−88.0	0	-	-	-
**5c_1_**·NO_3_^–^	−90.2	0	3.36(1.04) ^b^	8.71(4.72) ^b^	0.00(0.25) ^b^
**6c_1_**·NO_3_^–^	−88.1	0	2.14(1.75) ^b^	6.45(5.20) ^b^	0.18(0.15) ^b^
**7c_1_**·NO_3_^–^	−88.6	1	2.10(1.82) ^b^	6.33(5.03) ^b^	0.18(0.14) ^b^
**5c_2_**·NO_3_^– b^	−84.2	0	0.92	4.80	0.25
**6c_2_**·NO_3_^– b^	−85.4	2	1.83	5.70	0.16
**7c_2_**·NO_3_^– b^	−88.7	2	2.13	6.10	0.12
**8c_2_**·NO_3_^–^	−94.4	0	-	-	-
**9e**·NO_3_^–^	−80.7	2	-	-	-
**9c**·NO_3_^–^	−79.8	1	-	-	-
**10c**·NO_3_^–^	−82.3	0			
**10c_1_**·NO_3_^–^	−82.0	0	1.36(2.03) ^b^	4.48(5.66) ^b^	0.18(0.11) ^b^
**11c_1_**·NO_3_^–^	−82.3	0	1.22(2.33) ^b^	4.01(5.97) ^b^	0.17(0.08) ^b^
**12c_1_**·NO_3_^–^	−83.8	0	-	-	-
**9d**·NO_3_^–^	−79.7	2	-	-	-
**10d**·NO_3_^–^	−78.6	0	-	-	-
**11d**·NO_3_^–^	−78.3	0	-	-	-
**12d**·NO_3_^–^	−76.8	0	-	-	-

^a^ Atoms in molecules (AIM) properties computed at the BCP resulting from the O_2_NO^–^···C5 contact. ^b^ AIM properties computed at the BCP resulting from the anion···X contact.

As in Cl^–^ complexes, hydrogen bond-based **d** complexes could not be found for **1**–**8**. From the geometries included in Figure 6, we observe that **9d**–**12d** complexes have the same recognition pattern, in which the anion is interacting with two H atoms in ortho to two phenyl rings, with the H atom at C2. The CH···ONO_2_^–^ distance oscillates from 2.14–2.41 Å for X = O to 2.28–2.55 Å for X = Te.

Finally, **e** complexes are characterized by showing intermolecular contacts with the C atoms of the heteroaromatic system and were obtained only for Compounds **5**–**9**. Thus, in **5e**–**8e** complexes, the anion is located parallel to and above the ring showing distances of 2.8–3.0 Å between the O atoms of nitrate and the ring plane. Two hydrogen bonds are also observed with the H atoms of C3 and C5 methyl groups. In Complex **9e**, the nitrate is located above the heteroaromatic ring and perpendicular to it, giving rise to three intermolecular contacts between one O atom of NO_3_^–^ and two C atoms (C1 and C5) and between a second NO_3_^–^ O atom and C3.

The values of the interaction energies of all complexes (Table 5) range from −109.0 kcal·mol^−1^ for **1b**·NO_3_^–^ to −76.8 kcal·mol^−1^ for **12d**·NO_3_^–^. The largest binding energies are found for **a** and **b** σ-complexes, with **a** complexes and hydrogen bond-based **d** complexes being the most and least favorable ones, respectively. Moreover, complexes where anion···X contacts are found (**c**) usually have larger binding energies than those where only C atoms are involved in the interaction with the anion (**e**). Substitution affects the binding energies the same way as in chloride complexes, *i.e*., unsubstituted complexes are the most favorable (∆*E* between −109.0 and −96.4 kcal·mol^−1^), followed by their trimethyl (∆*E* between −94.4 and −84.2 kcal·mol^−1^) and triphenyl derivatives (∆*E* between −83.8 and −76.8 kcal·mol^−1^).

The origin and nature of these interactions have been analyzed within the frames of NBO, AIM and SAPT theories for selected complexes. According to the NBO analysis, the interaction in **4c** is based on a charge donation from the lone pairs of an O atom of NO_3_^–^ to the vacant π* orbital of the Te–C5 bond, as previously observed for their chloride analogues, resulting in a π-hole chalcogen bond. Results for **2c** and **3c** complexes show some inconsistencies and therefore are not reported. In contrast, interaction in **5c_2_**–**8c_2_** complexes come from Lp(O_NO3_)→σ*(X–C5) donor-acceptor contributions, except for **5c_2_**, where contributions are based on charge transfer from the anion to H atoms of the methyl groups. Moreover, for this last complex, a very small contribution is found for the Lp(O_pyrylium_)→π*(N–O) donor-acceptor interaction. Thus, results for **6c_2_**–**8c_2_** suggest the existence of a σ-hole chalcogen bond. Different charge-transfer contributions appear in **5c_1_**–**7c_1_** complexes, where Lp(O)→π*(C1–C2) donor-acceptor interactions for **6c_1_** and **7c_1_** and donation from O lone pairs of NO_3_^–^ to empty p orbitals of C1 are dominant, which will be in agreement with the formation of an anion-π interaction.

**Figure 7:**
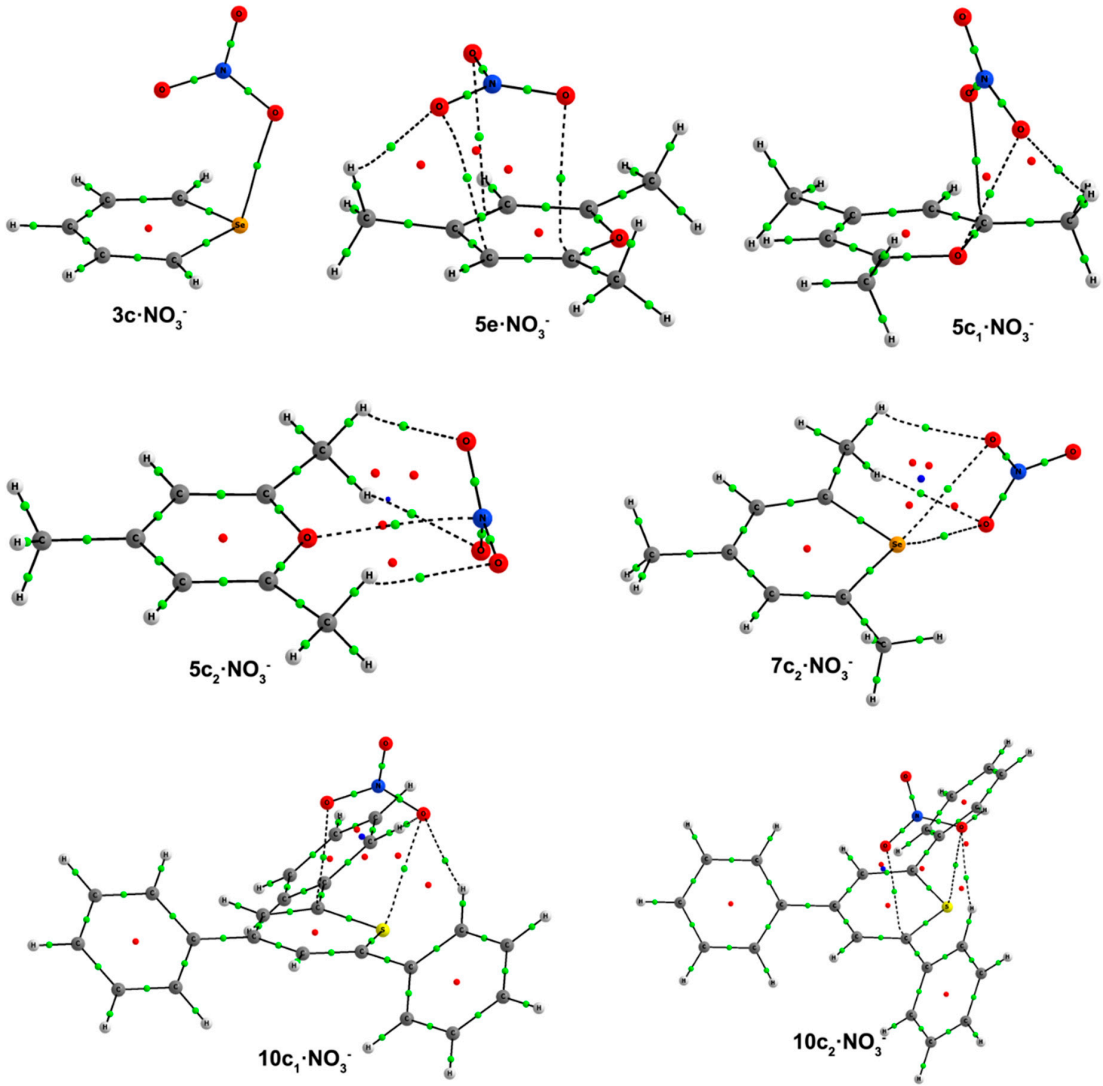
Molecular graphs of **3c**, **5e**, **5c_1_**, **5c_2_**, **7c_2_**, **10c_1_** and **10c_2_**·NO_3_^–^. The BCPs, ring and cage critical points are represented by green, red and blue dots, respectively. Only bond paths are depicted.

Consistent with the NBO results, AIM calculations for **c**·NO_3_^–^ complexes show the existence of one BCP connecting the X atom with ONO_2_^–^ (Figure 7). The values of the electron densities at these BCPs, comprised between 0.046 au for **3c**·NO_3_^–^ and 0.009 au for **5c_2_**·NO_3_^–^ (Table 5) and its positive values of the Laplacian are consistent with the existence of noncovalent interactions. However, the total energy densities are negative for **2c** and **3c**·NO_3_^–^ (−0.003 and −0.004 au, respectively), suggesting that these interactions have a small covalent character. In addition, according to the high electron density values (ρ = 0.2 au), and negative values of the Laplacian (between −0.40 and −0.24 au) and total energy densities (between −0.24 and −0.18 au) at the BCPs connecting the anion with the ring, **a**·NO_3_^–^ and **b**·NO_3_^–^ σ-complexes have a marked covalent character.

The SAPT analysis of complexes **2c**–**3c**·NO_3_^–^ and **11c_2_**–**12c_2_**·NO_3_^–^ (Table 4) shows similar conclusions to those obtained for chloride complexes. Indeed, the electrostatic component is the largest contributor ranging from −105.4 to −80.0 kcal·mol^−1^, and the smallest and largest contributions are found for X = O and X = Se, respectively. The dispersion contribution is practically the same for all complexes and the induction component varies according to the size of the chalcogen. Additionally, again, the consideration of δHF (Table 4) is very important at least for **2c**–**3c** compounds to reach an agreement between *E*_SAPT_ and ∆*E* values. In contrast, exchange, induction, dispersion and δHF contributions are very similar for **5e**–**7e**·NO_3_^–^ complexes, showing only small differences in the electrostatic component, which in the end could be responsible for the observed small differences in the binding energies.

### 2.4. Complexes with BF_4_^–^

**Figure 8:**
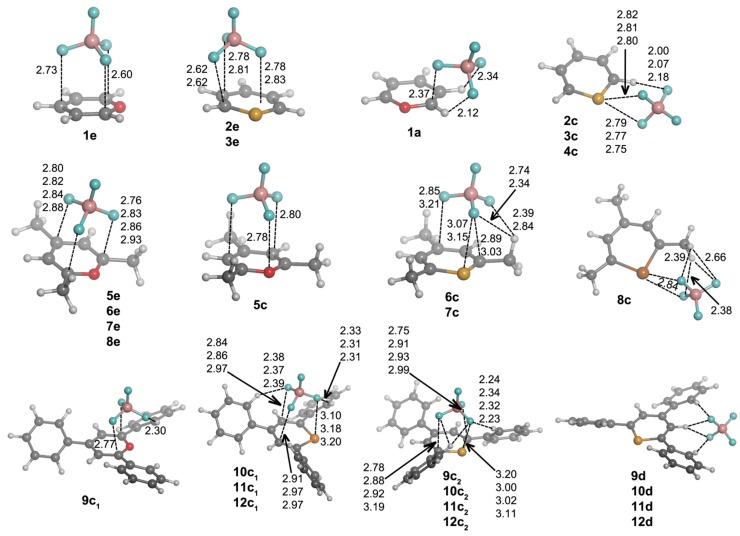
Optimized geometries of **a**·BF_4_^–^,**c**·BF_4_^–^, **d**·BF_4_^–^and **e**·BF_4_^–^ complexes. Selected distances (in angstroms) are shown.

In this third section, we are going to analyze the complexes formed by Compounds **1**–**12** with tetrafluoroborate as the anion. The exploration of the potential energy surface leads to the formation of four main structural configurations: Type **a**, **c**, **d** and **e** complexes, analogous to those previously described. The geometries of the complexes are shown in Figure 8, and their respective binding energies are listed in Table 6.

There is one Type **a** complex, **1a**, which exhibits a quite short F···C distance (2.37 Å) coupled with two hydrogen bonds with H atoms of C1 and C2. In **c**·BF_4_^–^ complexes, different orientations of tetrafluoroborate are observed. For unsubstituted compounds, BF_4_^–^ is located establishing two interactions of the F···X type and one hydrogen bond with the H atom at C2 in **2c**–**4c**·BF_4_^–^ (Figure 8). Type **c** complexes for the trimethyl derivatives show two different approximations of the anion. In **5c**–**7c**, the anion is placed above the ring with three F atoms pointing at the aromatic system. In **5c**, two F atoms are facing C2 and C4, and one F atom is facing the oxygen atom. The anion is slightly displaced towards the right half of the molecule in **6c**–**7c** with two F atoms giving rise to three contacts with C1, C3 and the X atom. In triphenyl **c_1_** complexes, two F atoms are directed at C2, C4 and a third F atom at X, whereas in **c_2_** complexes, two F atoms are directed at C1 and C5 and a third F atom at X (Figure 8), with hydrogen bonds with the H atoms in ortho to the C1 and C5 phenyl moieties. In Figure 8, we observe that **9d**–**12d** complexes have the same recognition pattern, in which the anion is interacting with two H atoms in ortho to two phenyl rings and with the H atom at C2. The CH···FBF_3_^–^ distance oscillates from 2.07–2.33 Å for X = O to 2.21–2.72 Å for X = Te. Lastly, **e** complexes have the anion located above the aromatic ring. As seen in previous BF_4_^–^ complexes, the most favorable disposition of the anion is that with three F atoms directed at the aromatic system. In fact, tetrafluoroborate is symmetrically placed, facing C1, C3 and C5 in **1e** and **5e**–**8e**·BF_4_^–^, and a little bit displaced to one side of the molecule in **2e**–**3e**·BF_4_^–^, as depicted in Figure 8, most likely due to some sort of interaction between an F atom and X = S, Se.

**Table 6:** Interaction energies (∆*E*, in kcal·mol^−1^), electron density (ρ_BCP_), its Laplacian (∇^2^ ρ_BCP_) and the total electron energy density (*H*_BCP_) for the BCPs of F_3_BF^–^···C contacts of complexes of Compounds **1**–**12** with BF_4_^–^ and the number of imaginary frequencies (Nimag).

Complex	∆*E*	Nimag	ρ_BCP_ × 10^2^	∇^2^ ρ_BCP_ × 10^2^	*H*_BCP_ × 10^2^
**1e**·BF_4_^– a^	−89.3	0	1.63(1.26)	6.28(4.94)	0.26(0.24)
**2e**·BF_4_^– b^	−85.8	0	1.64(1.11)	6.53(4.51)	0.28(0.22)
**3e**·BF_4_^– b^	−84.9	0	1.64(1.10)	6.54(4.66)	0.28(0.21)
**1a**·BF_4_^–^	−87.2	0	2.73	9.76	0.24
**2c**·BF_4_^–^	−84.7	0	1.59	6.30	0.25
**3c**·BF_4_^–^	−86.8	0	1.84	6.79	0.24
**4c**·BF_4_^–^	−90.5	0	-	-	-
**5e**·BF_4_^– a^	−85.6	0	1.17(1.07)	4.74(4.43)	0.23(0.22)
**6e**·BF_4_^– a^	−82.9	0	1.05(1.03)	4.32(4.25)	0.22(22)
**7e**·BF_4_^– a^	−82.3	0	1.02(1.00)	4.07(4.13)	0.20(0.21)
**8e**·BF_4_^–^	−81.1	0	-	-	-
**5c**·BF_4_^– b^	−84.9	0	1.05	5.52	0.30
**6c**·BF_4_^– b^	−82.1	0	1.05	4.28	0.22
**7c**·BF_4_^– b^	−81.4	0	0.99	3.61	0.18
**8c**·BF_4_^–^	−82.4	1	-	-	-
**9c_1_**·BF_4_^–^	−72.1	1	-	-	-
**10c_1_**·BF_4_^–^	−73.9	0	-	-	-
**11c_1_**·BF_4_^–^	−73.4	1	-	-	-
**12c_1_**·BF_4_^–^	−73.5	0	-	-	-
**9c_2_**·BF_4_^–^	−73.6	2	1.20(0.43) ^b^	4.82(1.97) ^b^	0.23(0.12) ^b^
**10c_2_**·BF_4_^–^	−74.8	0	0.95(1.18) ^b^	3.77(4.37) ^b^	0.19(0.20) ^b^
**11c_2_**·BF_4_^–^	−74.1	0	0.89(1.28) ^b^	3.49(4.38) ^b^	0.18(0.19) ^b^
**12c_2_**·BF_4_^–^	−74.1	0	-	-	-
**9d**·BF_4_^–^	−73.8	1	-	-	-
**10d**·BF_4_^–^	−72.5	0	-	-	-
**11d**·BF_4_^–^	−72.0	0	-	-	-
**12d**·BF_4_^–^	−70.8	0	-	-	-

^a^ AIM properties computed at the BCP resulting from the F_3_BF···C3 contact. ^b^ AIM properties computed at the BCP resulting from the anion···X contact.

The values of the interaction energies are the smallest of all of the anion series (Table 6), ranging from −90.5 kcal·mol^−1^ for **4c**·BF_4_^–^ to −70.8 kcal·mol^−1^ for **12d**·BF_4_^–^. The largest binding energies are found for the unsubstituted complexes, and **d** complexes are the least favorable ones. Substitution affects the binding energies the same way as in chloride and nitrate complexes.

The NBO analysis of BF4^–^ complexes offers interesting results. In **2c**–**4c** complexes, the interaction is mainly based on charge donation from the lone pairs of an F atom of BF_4_^–^ to the empty σ* orbital of the X–C1 bond (σ-hole chalcogen bond) and from Lp(F)→σ*(C1–H) to a minor extent, except for **4c**, where the latter charge donation is dominant. A different picture is observed for **5c**-**8c** complexes with several contributions arising from Lp(F)→p(C1/C3) in **5c**, Lp(F)→p(C3/C4) in **6c** and Lp(F)→p(C5) in **7c** (in line with the formation of anion-π interactions). The Lp(F)→π*(X–C1) donor-acceptor contribution is common for these complexes, increasing its importance with the size of the chalcogen in such a way that it becomes the only contribution in **8c**. Contributions for **e** complexes are also based on charge transfer from lone pairs of F atoms to vacant p orbitals of C3 and C5 (and C1 for **5e**), consistent with the existence of an anion-π interaction. Moreover, **6e**–**8e** complexes have charge-transfer contributions of the Lp(F)→π*(X–C1) π-hole chalcogen bond type.

**Figure 9:**
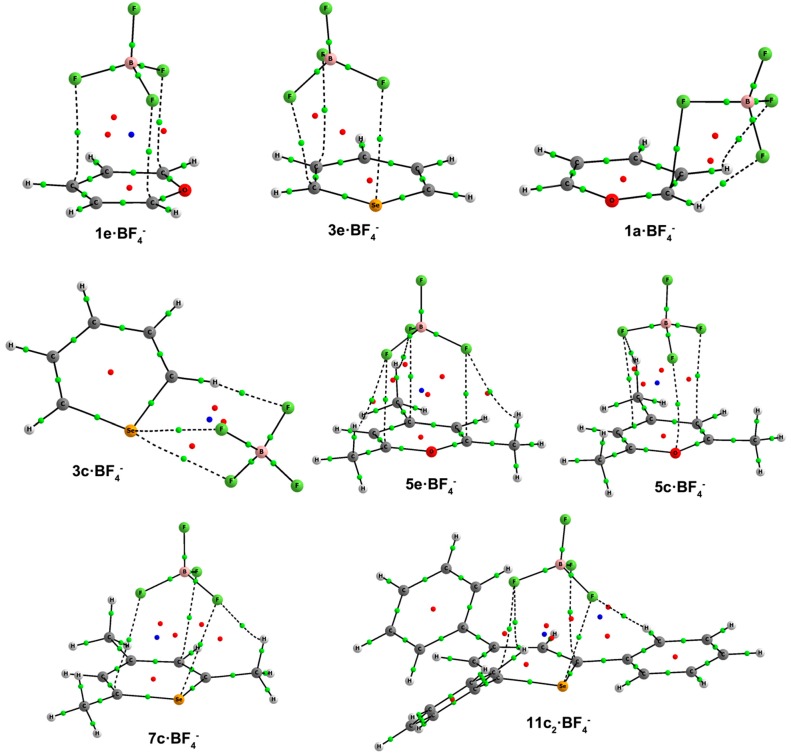
Molecular graphs of **1a**, **3c**, **5c**, **7c**, **11c_2_**, **1e**, **3e** and **5e**·BF_4_^–^. The BCPs, ring and cage critical points are represented by green, red and blue dots, respectively. Only bond paths are depicted.

AIM calculations for all complexes are in agreement with NBO results and show the existence of one BCP connecting the X, C and H atoms with FBF_3_^–^ (Figure 9). It is worth noting that the type of contacts with X (characteristic for **c** complexes) are also present in **2e**–**3e**·BF_4_^–^. The values of the electron densities (between 0.027 and 0.004 au), Laplacian (between 0.098 and 0.020 au) and total energy densities (between 0.003 and 0.001 au) at the BCPs of all complexes (Table 6) are consistent with the existence of noncovalent interactions.

The SAPT results for **1e**–**3e**, **5e**–**7e**, **1a**, **2c**–**3c** and **10c_2_**–**11c_2_**·BF_4_^–^ are analogous to the SAPT analysis of nitrate complexes. In general, all contributions are smaller than those for Cl^–^ and NO_3_^–^, which is reflected in the smallest binding energies of all series of anions. The largest contribution comes from electrostatics with values between −96.3 and −73.3 kcal·mol^−1^. Both dispersion and exchange contributions are kept more or less constant for all complexes, and the induction component only varies in c complexes.

### 2.5. Cambridge Structural Database Results

**Figure 10:**
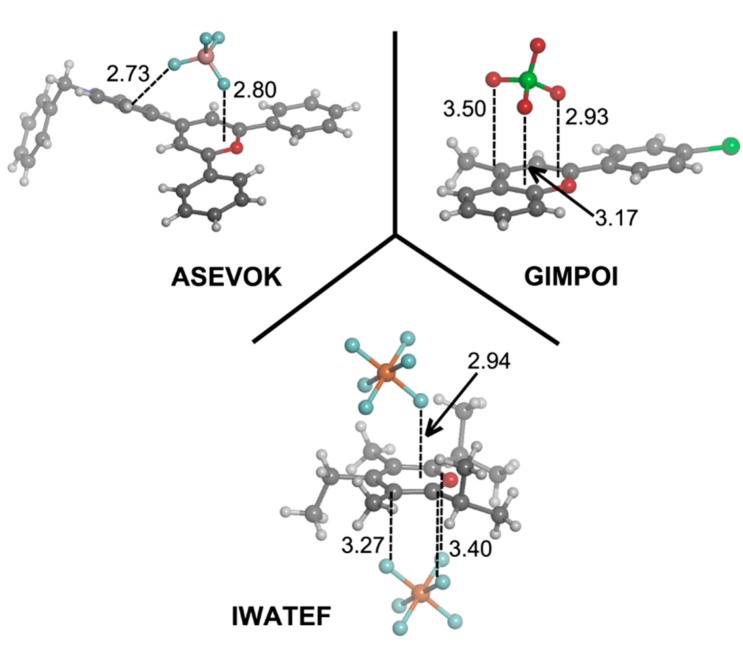
Fragments of the pyrylium-based X-ray crystal structures with ASEVOK, GIMPOI and IWATEF CSD reference codes. Distances in Angstroms.

**Figure 11:**
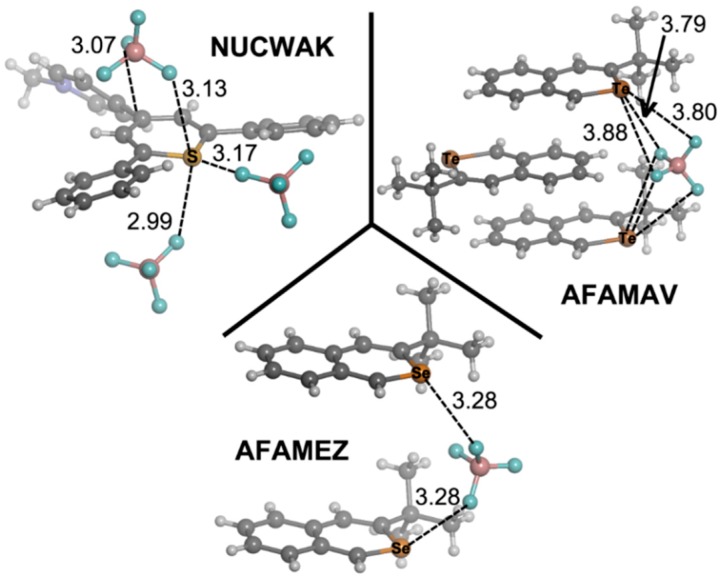
Fragments of the thiopyrylium- selenopyrylium- and telluropyrylium-based X-ray crystal structures with NUCWAK, AFAMEZ and AFAMAV CSD reference codes. Distances in Angstroms.

The Cambridge Structural Database (CSD) [37] is a convenient and reliable storehouse for geometric information. The utility of small molecule crystallography and the CSD in analyzing geometric parameters and nonbonding interactions is well established [38]. We have explored the CSD, searching for structures containing the pyrylium moiety **1**–**4** and we found 103 X-ray structures, 93 of them containing the pyrylium core. For thiopyrylium, we found eight structures and only one for both seleno- and telluro-pyrylium systems. In Table 7, we have gathered the number of structures for every family of compounds along with the occurrence of the associated anion. From the inspection of the results, we observe that the most common anion is perchlorate with 35 hits, followed by tetrafluoroborate (18 hits) and triflate (11 hits). We show in Figure 10 three pyrylium selected examples (CSD Reference Codes: ASEVOK [39], GIMPOI [40] and IWATEF) [41] and in Figure 11, three selected examples for the rest of the derivatives (CSD reference codes: NUCWAK [42], AFAMEZ [43] and AFAMAV) [43] that we have retrieved from the database, where the contacts between the anion and the cation have a strong influence in determining the crystal packing. In the ASEVOK structure, a BF_4_^–^ is directing one F atom to the heteroaromatic ring and a second one to an aromatic H atom. However, three O atoms of ClO_4_^–^ and three F atoms of PF_6_^–^ are facing the ring in GIMPOI and IWATEF, respectively, as previously shown in the computed **e**·BF_4_^–^ complexes. A good agreement is found between our computed complexes and the rest of the structures in Figure 11, where we observe several BF_4_^–^···X interactions for every example, as inferred from the values of the intermolecular distances. Particularly, in AFAMEZ and AFAMAZ, the anion is engaged in F_3_BF^–^···X interactions that act as a bridge joining two seleno- and telluro-pyrylium cationic molecules, giving rise to columns (Figure 11).

**Table 7:** Occurrence of pyrylium X-ray structures, retrieved from the Cambridge Structural Database (CSD), as a function of the anion.

Compound	Anion	Structures
**X = O**	ClO_4_^–^	33
	BF_4_^–^	15
	CF_3_SO_3_^–^	10
	PF_6_^–^	6
	I_3_^–^	3
	Cl^–^	2
	FeCl_4_^–^	2
	Br^–^	1
	CuCl_4_^2–^	1
	MnCl_4_^2–^	1
	Pd_2_Cl_6_^2–^	1
	ZnCl_4_^2–^	1
	SbCl_6_^–^	1
	HSO_4_^–^	1
	PhSO_3_^–^	1
	CCl_3_COO^–^	1
	Others	13
	**Total**	**93**
**X = S**	ClO_4_^–^	2
	SbCl_6_^–^	1
	BF_4_^–^	1
	CF_3_SO_3_^–^	1
	Others	3
	**Total**	**8**
**X = Se**	BF_4_^–^	1
**X = Te**	BF_4_^–^	1

## 3. Computational Methods

We carried out geometry optimizations by means of DFT calculations at the B97-D3 [44,45] level of theory. This approach is based on Becke’s generalized gradient approximation (GGA) functional introduced in 1997 [46] and is explicitly parameterized by including damped atom-pairwise dispersion corrections of the form *C*_6_ · *R*^−6^. We optimized the geometry of all compounds using restricted Kohn–Sham DFT [47]. In these calculations, the Ahlrichs augmented triple-ζ basis for all atoms (def2-TZVPD basis set, abbreviated as TZVPD) [48] was used, unless stated otherwise. Our DFT calculations were carried out using the resolution of the identity (RI) B97-D3, which uses an auxiliary fitting basis set [49] to avoid treating the complete set of two-electron repulsion integrals, thus speeding up calculations by a factor of 10. Because of the time-consuming nature of the calculations, we used the parallel RI-DFT [50] methodology. Geometries of all structures were optimized with the analytical gradient method without imposing any symmetry constraints, unless stated otherwise. The vibrational frequencies were calculated at the RI-BP86-D3/def2-TZVPD level of theory. For our preliminary calculations, we carried out geometry optimizations using the MP2 method (RI-MP2) [51,52,53] with the aug-cc-pVTZ basis set and refined the energies by using the coupled-cluster method with singles and doubles excitations and triple excitations non-interatively (CCSD(T)) with a complete basis set (CBS). The CCSD(T) technique provides reliable interaction energies only if they are combined with extended AO basis sets, and the larger the basis set, the better the interaction energies that result. Owing to the rather strong dependence of the interaction energy on the AO basis set size, it is recommended that the relevant calculations be performed at the complete basis set (CBS) limit. Different extrapolation schemes have been introduced, and the scheme of Helgaker *et al*. [54] has become the most widely used. Here, the HF and correlation (MP2) energies are extrapolated separately as follows:

(1) $$E_{X}^{HF}{=E}_{\text{CBS}}^{\text{HF}}+Ae^{-\propto \text{X}}$$

(2) $$E_{X}^{HF\;}=E_{\text{CBS}}^{\text{HF}}+Ae^{-\propto \text{X}}$$

(3) $$\text{Δ}E_{CBS}^{CCSD\left(T\right)}=E_{\text{CBS}}^{\text{MP2}}\text{ +}\;{\left(\text{Δ}E^{CCSD\left(T\right)}\;-\;\Delta E^{MP2}\right)}_{AVDZ}$$

where *E*_X_ and *E*_CBS_ are the energies for the basis set with the largest angular momentum *X* and for the complete basis set, respectively. The CCSD(T)/CBS level can be attained via a separate extrapolation of the MP2 and higher-order correlation energies towards the basis set limit (Equation (3)). Here, each of the components is differently sensitive to the AO basis set: the MP2 interaction energy is the more slowly converging, and the larger the basis set used in the extrapolation, the better. In our case, we have used from the aug-cc-pVTZ (AVTZ) to the aug-cc-pVQZ (AVQZ) basis sets. The second term, called the CCSD(T) correction term (∆CCSD(T)), is determined as the difference between the CCSD(T) and MP2 interaction energies and converges much faster than the previous term. The use of such a term is possible, because the MP2 and CCSD(T) interaction energies converge with basis set size in a very similar way; consequently, its difference is much less basis set dependent, and much smaller basis sets can be applied. In our case, we have used the AVDZ basis set to compute the CCSD(T) correction.

All of the theoretical calculations described herein were carried out by using TURBOMOLE Version 6.5 [55,56], apart from the CCSD(T) calculations, which were computed by using the MOLPRO program [57,58].

The partitioning of the interaction energies into the individual electrostatic, induction, dispersion and exchange-repulsion components was carried out by performing density functional theory (DFT) calculations combined with the symmetry-adapted perturbation theory (DFT-SAPT) approach [59,60,61,62,63] with the MOLPRO program. The DFT-SAPT total interaction energy (*E*_int_) is given in terms of the first-, second- and higher-order correction interaction terms that are indicated by the superscripts in Equation (4):

(4) $$E_{\text{int }}=E_{\text{el}}^{\text{1}}{\text{ + E}}_{\text{exch }}^{\text{1}}+E_{\text{ind }}^{\text{2}}+E_{\text{ind-exch}}^{\text{2}}+E_{\text{disp }}^{\text{2}}+E_{\text{disp-exch}}^{\text{2}}\text{ + δHF}$$

where $E_{\text{el}}^{\text{1}}$ and $E_{\text{exch}}^{\text{1}}$ are the sum of the electrostatic interaction energy and the first-order exchange energy, respectively. $E_{\text{ind}}^{\text{2}}$, $E_{\text{ind-exch}}^{\text{2}}$, $E_{\text{disp}}^{\text{2}}$ and $E_{\text{disp-exch}}^{\text{2}}$ denote the induction (with response) energy, the second order induction-exchange (with response) energy, the dispersion energy and the exchange-dispersion contribution, respectively. The Hartree–Fock (HF) correction for third- and higher order contributions to the intermolecular interaction potential (δHF) is not included in DFT-SAPT calculations and can be estimated as the difference between the supermolecular HF energy and the sum of electrostatic, induction and their exchange counterparts obtained at the HF level. The aug-cc-pVDZ basis set was used to compute this correction apart from H, for which we used the cc-pVDZ basis set. For brevity, we will often refer to the aug-cc-pVXZ by the shorthand notation AVXZ. Physically meaningful separation of the interaction energy may be obtained by classifying the cross terms induction-exchange $E_{\text{ind-exch}}^{\text{2}}$ and dispersion-exchange $E_{\text{disp-exch}}^{\text{2}}$ as a part of the induction and the dispersion, respectively [64,65]. The AVTZ basis set was used for the DF-DFT-SAPT calculations. As the auxiliary fitting basis set, the JK-fitting basis of Weigend [66] was employed. The cc-pVQZ JK-fitting basis was used for all atoms. For the intermolecular correlation terms, *i.e*., the dispersion and exchange-dispersion terms, the related AVTZ MP2-fitting basis of Weigend, Köhn and Hättig [67] was employed. In the DFT-SAPT calculations, the BP86 functional (the B88 exchange functional [68] in combination with the P86 gradient correction) [69] was employed using the B97-D3/def2-TZVPD optimized geometries.

The electronic properties of the systems have been analyzed within the atoms in molecules (AIM) theory [70,71] by using the AIMAll [72] program. The AIM theory is based on the topological analysis of the electron density, ρ(r). The presence of a path linking two nuclei in an equilibrium structure implies that the two atoms are bonded to one another, and it is characterized by the bond critical point (BCP). Topologically, a BCP corresponds to a point in the real space where the gradient of the density, ∇ ρ_BCP_, is zero and where the curvature of ρ_BCP_, expressed through the three eigenvalues of the diagonalized Hessian of ρ_BCP_, is positive for an eigenvector linking two atomic centers and negative for the two others perpendicular to it. A chemical bond thus results from the competition of the parallel expansion of ρ, which separates charges in their respective atomic basins and the perpendicular contraction of ρ toward a bond path. The dominant effect is measured by the Laplacian of ρ_BCP_, ∇^2^ ρ_BCP_. Values of ρ_BCP_ greater than 0.2 are typical of covalent bonds, and ∇^2^ ρ_BCP_ is generally less than zero for such interactions, reflecting the concentration of electron density along the bond path linking the bonded atoms. Thus, when ∇^2^ ρ_BCP_ < 0, charge is concentrated at the critical point, while when ∇^2^ ρ_BCP_ > 0, charge is locally depleted. Additional information of the chemical bond can be obtained by applying the local expression of the virial theorem to the critical point, 1/2∇^2^ρ_BCP_ = 2*G*_BCP_ + *V*_BCP_, where *G*_BCP_ and *V*_BCP_ are the kinetic and potential energy densities at the BCP, respectively [73]. Cremer and Kraka demonstrated that the sign of the total energy, *H*_BCP_ = *G*_BCP_ + *V*_BCP_, is an index of the amount of covalency in the chemical interactions [73,74]. *H*_BCP_ is negative for interactions with significant sharing of electrons. The remaining two stable critical points occur as a consequence of particular geometrical arrangements of bond paths, and they define the remaining elements of molecular structure, *i.e*., rings (ring critical point, RCP) and cages (cage critical point, CCP).

The bonding features of all of the studied molecules and the net atomic charges were also analyzed by means of the natural bond orbital (NBO) and the natural population analysis (NPA) [75,76]. For such purposes, the NBO 3.1 program [77] has been used. The donor-acceptor interactions are quantified by examining all possible interactions between filled (donor) Lewis-type NBOs and empty (acceptor) non-Lewis NBOs and by evaluating their energetic importance by using the second-order perturbation theory in the NBO basis [78]. When interpreting the results of such estimations, it should be noted that this approach is only performed at the SCF level of theory (*i.e*., the Fock or Kohn−Sham operator is analyzed in the basis of the NBO), and only bonding interactions are considered (*i.e*., antibonding contributions are not covered by the NBO analysis and must be calculated separately). Because these interactions lead to the loss of occupancy from localized NBOs of an idealized Lewis structure to empty non-Lewis orbitals (and thus, to departure from an idealized Lewis structure description), they are referred to as delocalization corrections to the zeroth-order natural Lewis structure.

## 4. Conclusions

In summary, we have examined the molecular recognition in pyrylium, thio-, seleno- and telluro-pyrylium salts where anions of different morphologies were chosen, as well as different substitution of the heteroaromatic ring. The binding energy for the series of anions follows the ordering ∆*E*_Cl_ > ∆*E*_NO3_ > ∆*E*_BF4_, and within every family of compounds, ∆*E*_X_ > ∆*E*_X-Me3_ > ∆*E*_X-Phe3_. In general, for every derivative, the most favorable **a** and **b** complexes are found for X = O. Conversely, the largest binding energies in c complexes are obtained for the most electrophilic X = Te. Complexes strictly based on hydrogen-bonding interactions (**d** complexes) are the least favorable. For the monoatomic chloride anion, σ-complexes are generally the most favorable structures, except for triphenyl and X = Te trimethyl complexes, where the interactions of the anion with the chalcogen atom are important. The trigonal planar nitrate σ-complexes have the largest binding energies for the unsubstituted compounds. However, for the trimethyl and triphenyl derivatives, other configurations are more important, with the O atoms of nitrate either anion-π interacting with the ring C atoms (**e** complexes) or establishing interactions with the chalcogen atoms (**c** complexes) by means of σ- and π-hole chalcogen bonds. For the tetrahedral tetrafluoroborate anion, σ-complexes could not be found, and as in nitrate, **e** and **c** complexes are predominant. These complexes present multiple intermolecular contacts (anion-π interactions and σ- and π-hole chalcogen bonds) of the F···C, F···X and F···HC type, usually resulting from the interaction of three F atoms of a single BF_4_^–^ molecule with the cation. The interaction in all complexes is dominated by electrostatics. However, the induction term is especially important in chloride complexes. Finally, by analyzing the CSD, we have found several X-ray structures in which both anion-π interactions and chalcogen bonds are present, giving consistency to the computational study.
